# Tissue Doppler imaging is a sensitive echocardiographic technique to detect subclinical systolic and diastolic dysfunction of both ventricles in type 1 diabetes mellitus

**DOI:** 10.1186/s12872-016-0242-2

**Published:** 2016-04-22

**Authors:** David Suran, Andreja Sinkovic, Franjo Naji

**Affiliations:** Department of Cardiology and Angiology, Clinic for Internal Medicine, University Medical Centre Maribor, Ljubljanska ulica 5, 2000 Maribor, Slovenia; Department of Medical Intensive Care, Clinic for Internal Medicine, University Medical Centre Maribor, Ljubljanska ulica 5, 2000 Maribor, Slovenia

**Keywords:** Diabetes mellitus type 1, Left ventricular dysfunction, Right ventricular dysfunction, Tissue Doppler imaging

## Abstract

**Background:**

Subclinical left (LV) and right ventricular (RV) dysfunction has been demonstrated in type 2 diabetes mellitus and evidence indicates impaired LV diastolic function in type 1 diabetes mellitus (T1DM) as well. The aim of our study was to evaluate the role of tissue Doppler imaging (TDI) in assessment of global LV and RV function in T1DM patients.

**Methods:**

A detailed two-dimensional, pulsed wave Doppler and pulsed wave TDI analysis was performed in 53 normotensive middle-aged T1DM patients and compared to healthy controls.

**Results:**

In T1DM patients TDI analysis revealed reduced mean mitral septal and lateral E’ velocities as well as reduced mean tricuspid E˙t velocity compared to healthy controls (E’sept 8.89 ± 1.89 cm/s vs. 11.50 ± 2.41 cm/s, *p* < 0.001; E’lat 12.29 ± 2.58 cm/s vs.15.30 ± 2.95 cm/s, *p* < 0,001; E’t 13.56 ± 2.91 cm/s vs. 15.60 ± 2.99 cm/s, *p* = 0.001). Mean ratios E/E’sept, E/E’lat and E/E’t were significantly higher in diabetics with cutoff value of 7.4 for E/E’sept and 3.4 for E/E’t, differentiating diabetics with LV and RV diastolic impairement from matched healthy controls (sensitivity 76.5 %, specificity 73.8 % for E/E’sept and sensitivity 72.1 %, specificity 66.7 % for E/E’t). Myocardial acceleration during isovolumetric contraction (IVA) measured at the septal mitral (LV IVA) and lateral tricuspid annulus (RV IVA) was the only parameter indicating reduced contractility of both ventricles in diabetics compared to controls (LV IVA 230.70 ± 61.26 cm/s^2^ vs. 283.32 ± 59.74 cm/s^2^, *p* < 0,001; RV IVA 275.48 ± 68.08 cm/s^2^ vs. 316.86 ± 80.95 cm/s^2^, *p* = 0.011). LV IVA had better diagnostic accuracy than RV IVA to predict early contractile impairement in T1DM patients (area under the curve 0.758, *p* < 0.001 for LV IVA and 0.648, *p* = 0.017 for RV IVA).

**Conclusions:**

TDI is essential to detect subclinical diastolic deterioration of both ventricles in T1DM patients. TDI-derived IVA might be useful to assess early systolic alterations of both ventricles in T1DM patients.

## Background

Patients with diabetes mellitus (DM) have high incidence of heart failure [1, 2]. DM promotes myocardial damage even in the absence of hypertension, valvular or ischemic heart disease and the condition has been characterised as diabetic cardiomyopathy [1–3].

Most echocardiographic studies have been performed in populations with type 2 diabetes mellitus (T2DM), while in type 1 DM (T1DM) patients the developement of left ventricular (LV) and right ventricular (RV) dysfunction has been much less studied [4–6]. Recently, a large observational study has documented a high degree of LV diastolic dysfunction in T1DM patients, while data on RV diastolic dysfunction in this population are limited [7]. Impact of T1DM on development of LV and RV systolic dysfunction is controversial – very recent tissue deformation studies revealed some evidence of LV and RV contractile impairement, while the majority of prior studies using conventional echocardiographic parameters or even tissue Doppler imaging (TDI) did not show the difference [8–12].

The objective of the present study was to evaluate the potential role of TDI in detection of subclinical global LV and RV dysfunction in normotensive T1DM patients without other comorbidities.

## Methods

### Study design and participants

This was a cross-sectional study, conducted in the Functional Diagnostic Unit of the Department of Cardiology and Angiology at University Medical Centre Maribor, Slovenia. The study was approved by the National Medical Ethics Committee of the Republic of Slovenia (Medical Ethics Committee approval number 130/10/13). Written informed consent was obtained from all the participants. The study protocol conformed to the ethical guidelines of the Declaration of Helsinki.

We enrolled 53 normotensive patients with T1DM known for more than 5 years (mean age 44.3 ± 5.4 years; 56.6 % men, 43.4 % women). Patients were asymptomatic at enrollement and in the past; they had no history of heart failure or coronary artery disease. They were all in sinus rhythm.

The control group consisted of 48 healthy volunteers with mean age of 42.7 ± 6.1 years (56.3 % men and 43.7 % women). All patients and healthy subjects were recruited on the outpatient basis.

Before enrollment all medical records of included diabetic patients and controls were verified and clinical history obtained. Smoking was documented in all participants. Detailed clinical examination was performed in all subjects, including measurements of blood pressure twice in supine position, body height and weight to calculate body mass index (BMI). Electrocardiogram (ECG) was recorded in supine position.

Exclusion criteria were age above 53 and below 33 years, arterial hypertension, duration of T1DM less than 5 years, ischaemic heart disease (evaluated by careful consideration of medical history, ECG, echocardiographic assessment of regional LV wall motion abnormalities), overt left ventricular systolic dysfunction-left ventricular ejection fraction (LVEF) < 50 %, bundle branch block, more than mild valvular regurgitations and valvular stenosis of any degree, cardiomyopathies (dilatative, hypertrophic, restrictive), congenital heart diseases, arrhythmias, chronic pulmonary disease, pulmonary hypertension, untreated thyroid disease, systemic connective tissue diseases, pregnancy, extreme obesity (BMI above 35), advanced diabetic nephropathy (glomerular filtration rate below 60 ml/min), low quality echocardiographic image. The same exclusion criteria were used for both groups.

### Laboratory measurements

Before echocardiographic examination blood samples were drawn in patients with T1DM to measure serum creatinine, lipid profile and glycosylated hemoglobin (HbA1c). In addition, all results of HbA1c measurements in the last 5 years were collected – HbA1c measurements were performed in the diabetes outpatient clinic of our institution at regular 6-month follow-up.

Serum creatinine was measured by IDMS-traceable enzymatic assay (Siemens Healthcare Diagnostics Inc., Newark, USA; normal levels 44–97 μmol/l) [13].

The lipid profile consisted of serum triglycerides, total serum cholesterol, LDL and HDL cholesterol. Triglycerides were measured the by the enzymatic method (Siemens Healthcare Diagnostics Inc., Newark, USA; normal levels 0.6–1.7 mmol/L) and total serum cholesterol by the cholesterol esterase enzymatic assay (Siemens HealthCare Diagnostics Inc., Newark, USA); high density lipoproteins (HDL) and low density lipoproteins (LDL) were measured by the homogenous direct method (Siemens Healthcare Diagnostics Inc., Newark, USA) [13].

HbA1c was measured by the ion-exchange high performance liquid chromatography (Bio-Rad Laboratories, USA; normal levels 4.0–6.0 %) [13].

The degree of albuminuria was assessed in the second morning urine sample by the nephelometric method (Siemens HealthCare Diagnostics Inc., Newark, USA) and expressed as urine albumin-to-creatinine ratio (UACR, normal levels below 30 mg albumin/g creatinine). Albuminuria was defined as UACR ≥ 30 mg albumin/g creatinine [13].

### Echocardiography

All echocardiographic examinations were performed by a single experienced echocardiographer on the same ultrasound machine (iE33, Philips Medical Systems) at the same institution. A detailed two-dimensional (2D), pulsed wave Doppler (PWD) and pulsed wave TDI (pwTDI) analysis of the LV and RV were performed. Color Doppler recordings were obtained to exclude valvular dysfunction. All measurements were performed on the basis of the American Society of Echocardiography and European Association of Cardiovascular Imaging recommendations [14–16].

The maximal end-systolic volume of the left atrium (LA) was measured by the 2D biplane area-length formula and indexed to body surface area (BSA). Right atrial (RA) area was measured in four-chamber view and indexed to BSA. Modified biplane Simpson’s method was used to determine LVEF.

Mitral and tricuspid inflow velocities were evaluated by PWD from the apical four-chamber view and the following parameters were measured: peak early mitral inflow velocity (E), peak late mitral inflow velocity (A), E/A ratio, deceleration time of the E velocity (DT). From the tricuspid inflow pattern we measured peak early (Et) and late (At) diastolic velocity, Et/At ratio and deceleration time (DT-t).

PwTDI recordings were acquired from the apical four-chamber view and were used to measure peak myocardial systolic an diastolic velocities at the septal (S’sept, E’sept, A’sept) and lateral corner (S’lat, E’lat, A’lat) of the mitral annulus and peak myocardial systolic and diastolic velocities at the lateral tricuspid annulus (S’t, E’t, A’t). PwTDI-derived peak myocardial velocity during isovolumetric contraction (IVV) (cm/s) was measured at the septal mitral (LV IVV) and lateral tricuspid annulus (RV IVV). Myocardial acceleration during isovolumetric contraction (IVA) (cm/s^2^) was calculated for both ventricles (LV IVA and RV IVA) as the ratio of IVV devided by the acceleration time (AT), which was defined as the time spent from baseline to the peak velocity of isovolumetric contraction.

The LV and RV myocardial performance index (MPI) was assessed by pwTDI and calculated as the sum of the isovolumetric contraction time and isovolumetric relaxation time divided by ventricular ejection time.

All Doppler parameters except IVV and AT were recorded at a sweep speed of 75 mm/s at end expiration, while IVV and AT were recorded at a speed of 150 mm/s. The average value of three consecutive cardiac cycles was considered in statistical analysis. Settings were adjusted for a frame rate of above 150 Hz.

### Statistical analysis

SPSS Statistics 22.0 (IBM Corp., Armonk, New York, USA) was used for statistical analysis. The Shapiro–Wilks test was used to confirm normal distribution of data for all variables. Categorical variables were presented as percentages and compared with two-tailed Fisher’s exact test. Normally distributed continuous variables were presented as mean ± standard deviation and compared with unpaired Student’s t-test. The correlations between continuous variables were assessed with the Pearson’s correlation coefficient (r). *P* < 0.05 was considered statistically significant. Also, we performed receiver operating characteristic (ROC) curve analysis of LV IVA, LVEF, RV IVA, E/E’sept, E/E’t, LA volume index and RA area index to select optimal cutoff values based on equally balanced sensitivity and specificity to detect early LV and RV impairement in diabetic patients.

To assess intraobserver variability a TDI dataset of 16 randomly selected participants was analyzed for LV IVV, LV IVA, RV IVV and RV IVA by the same observer on two different days. For interobserver variability assessment a second observer blinded to the clinical information and to the results of the first observer analyzed the same TDI recordings. Variability was calculated in two ways: a) as the mean percent error, derived as the difference between two measurements, divided by the mean value of these two measurements and b) by intraclass correlation coefficients (ICC) for absolute agreement.

## Results

### Clinical characteristics and laboratory data

Ninety-seven T1DM patients treated at our diabetes outpatient clinic met inclusion criteria and were invited to participate in the study. Thirty-four patients refused to join the study and ten patients were later excluded because of poor echocardiographic image quality or valvular abnormalities. Finally, fifty-three T1DM patients were enrolled to the study.

Demographic and clinical data are presented in Table 1. There was no difference in age, gender, BSA, BMI, blood pressure and smoking between the two groups. Mean diabetes duration was 18.2 ± 7.2 years. All diabetic patients were treated with insulin; 45.3 % of patients used an insulin pump and 44.7 % of them were on standard insulin regimen.

**Table 1:** Demographic and clinical data of diabetic patients and control subjects

Variable	Diabetic patients (*n* = 53)	Healthy controls (*n* = 48)	*p*-value
Age (years)	44.3 ± 5.4	42.7 ± 6.1	0.159
Gender (male/female, %)	56.6/43.4	56.3/43.7	0.873
Body mass index (kg/m2)	27.0 ± 4.0	25.6 ± 3.5	0.068
Body surface area (m^2^)	1.94 ± 0.2	1.90 ± 0.2	0.430
Smoking (%)	24.5	33.3	0.454
Systolic blood pressure (mmHg)	126.0 ± 9.5	123.0 ± 10.7	0.168
Diastolic blood pressure (mmHg)	79.5 ± 5.8	77.0 ± 7.0	0.061

Laboratory data are presented in Table 2. Mean HbA1c level was 7.6 ± 1.1 at the time of enrollment. Mean value of HbA1c in the last 5 years was 7.2 ± 0.8. Serum creatinine was normal in all patients. Albuminuria was detected in only one patient (1.9 %) and mean UACR was 6.5 ± 5.4.

**Table 2:** Laboratory data of diabetic patients (*n* = 53)

Variable	Mean ± SD
HbA1c (at enrollment, %)	7.6 ± 1.1
HbA1c (5-year average, %)	7.2 ± 0.8
Serum creatinine (μmol/L)	69.6 ± 12.6
UACR (mg albumin/g creatinine)	6.5 ± 5.4
Total cholesterol (mmol/L)	4.9 ± 0.8
HDL (mmol/L)	1.8 ± 0.5
LDL (mmol/L)	2.9 ± 0.8
Triglycerides	0.9 ± 0.5

*SD* standard deviation, *HbA1c* glycosylated hemoglobin, *UACR* urinary albumin-to-creatinine ratio, *HDL* high density lipoprotein, *LDL* low density lipoprotein

### Echocardiography data

Standard echocardiographic measurements, PWD and TDI-derived data are summarised in Table 3. In T1DM patients in comparison to healthy controls we observed significantly increased LA volume index and RA area index (Table 3).

**Table 3:** Echocardiographic data of diabetic and control subjects

	Diabetic partients (*n* = 53)	Healthy controls (*n* = 48)	*p*-value
LA/RA dimensions (mean ± SD)			
LA volume (ml)	57.78 ± 11.15	43.89 ± 7.97	<0.001
LA volume/BSA (ml/m^2^)	29.44 ± 3.82	22.94 ± 3.25	<0.001
RA area (cm^2^)	15.38 ± 2.15	12.69 ± 2.05	<0.001
RA area/BSA (cm^2^/m^2^)	7.90 ± 0.95	6.61 ± 0.79	<0.001
LV dimensions (2D), LVEF (mean ± SD)			
IVS (cm)	0.92 ± 0.11	0.82 ± 0.09	<0.001
LVIDd (cm)	4.61 ± 0.39	4.65 ± 0.42	0.638
LVIDd/BSA (cm/m^2^)	2.38 ± 0.24	2.46 ± 0.21	0.106
LVPW (cm)	0.93 ± 0.11	0.85 ± 0.09	<0.001
LVEF (%)	66.57 ± 5.89	67.82 ± 5.08	0.324
RV dimensions (2D) and TAPSE (mean ± SD)			
RVD1 (cm)	3.49 ± 0.37	3.53 ± 0.94	0.786
RVD1/BSA (cm/m^2^)	1.79 ± 0.21	1.87 ± 0.44	0.262
RVD2 (cm)	2.72 ± 0.43	2.60 ± 0.38	0.211
RVD3 (cm)	7.10 ± 0.88	7.1 ± 0.82	0.977
TAPSE (cm)	2.73 ± 0.36	2.83 ± 0.26	0.154
Mitral PWD parameters (mean ± SD)			
E (m/s)	0.79 ± 0.16	0.74 ± 0.12	0.111
A (m/s)	0.68 ± 0.13	0.58 ± 0.10	<0.001
E/A	1.19 ± 0.25	1.33 ± 0.27	0.011
DT (ms)	187.76 ± 45.1	163.56 ± 28.14	0.002
Tricuspid (t) PWD parameters (mean ± SD)			
Et (m/s)	0.52 ± 0.11	0.49 ± 0.07	0.208
At (m/s)	0.44 ± 0.22	0.35 ± 0.06	0.007
Et/At	1.27 ± 0.31	1.41 ± 0.24	0.015
DT-t (ms)	214.26 ± 65.41	210.61 ± 48.87	0.796
LV TDI parameters (mean ± SD)			
S’ sept (cm/s)	8.46 ± 1.64	9.19 ± 1.19	0.012
S’ lat (cm/s)	10.62 ± 2.23	11.22 ± 2.29	0.183
E’ sept (cm/s)	8.89 ± 1.89	11.50 ± 2.41	<0.001
A’ sept (cm/s)	9.74 ± 1.95	9.41 ± 1.68	0.378
E’/A’ sept	0.94 ± 0.26	1.28 ± 0.42	<0.001
E/E’sept	9.07 ± 1.86	6.65 ± 1.35	<0.001
E’ lat (cm/s)	12.29 ± 2.58	15.30 ± 2.95	<0.001
A’ lat (cm/s)	9.64 ± 1.97	9.17 ± 2.45	0.296
E’/A’ lat	1.35 ± 0.48	1.81 ± 0.64	<0.001
E/E’ lat	6.55 ± 1.16	4.96 ± 0.90	<0.001
LV IVV (cm/s)	6.82 ± 2.22	7.18 ± 2.00	0.406
LV IVA (cm/s^2^)	230.70 ± 61.26	283.32 ± 59.74	<0.001
LV MPI	0.51 ± 0.10	0.49 ± 0.09	0.455
RV TDI parameters (mean ± SD)			
S’t (cm/s)	14.40 ± 2.50	14.38 ± 2.15	0.961
E’t (cm/s)	13.56 ± 2.91	15.60 ± 2.99	0.001
A’t (cm/s)	14.03 ± 3.59	12.09 ± 2.66	0.003
E’/A’t	1.01 ± 0.26	1.35 ± 0.39	<0.001
E/E’t	3.93 ± 1.04	3.27 ± 0.57	<0.001
RV IVV (cm/s)	10.82 ± 2.62	11.98 ± 2.81	0.045
RV IVA (cm/s^2^)	275.48 ± 68.08	316.86 ± 80.95	0.011
RV MPI	0.31 ± 0.07	0.29 ± 0.07	0.335

*LA* left atrium, *RA* right atrium, *BSA* body surface area, *LV* left ventricle, *LVEF* left ventricular ejection fraction, *IVS* interventricular septum, *LVIDd* left ventricular internal dimension at end diastole, *LVPW* left ventricular posterior wall thickness, *RV* right ventricle, *TAPSE* tricuspid anular plane systolic excursion, *RVD1* right ventricular basal diameter, *RVD2* right ventricular mid-cavity diameter, *RVD3* right ventricular longitudinal diameter; *E* peak early mitral filling velocity, *A* peak late mitral filling velocity, *DT* peak early mitral velocity deceleration time, *Et* peak early tricuspid filling velocity, *At* peak late tricuspid filling velocity, *DT-t* peak early tricuspid velocity deceleration time, *S’sept* peak systolic mitral annular velocity at the septal part of mitral annulus, *S’lat* peak systolic mitral annular velocity at the lateral part of mitral annulus, *E’sept* peak early diastolic mitral annular velocity at the septal part of mitral annulus, *E’lat* peak early diastolic mitral annular velocity at the lateral part of mitral annulus, *A’sept* peak late diastolic mitral annular velocity at the septal part of mitral annulus, *A’lat* peak late diastolic mitral annular velocity at the lateral part of mitral annulus, *S’t* peak systolic tricuspid annular velocity, *E’t* peak early diastolic tricuspid annular velocity, *A’t* peak late diastolic tricuspid annular velocity, *IVV* peak myocardial velocity during isovolumetric contraction, *IVA* myocardial acceleration during isovolumetric contraction, *MPI* myocardial performance index

Regarding LV echocardiographic measurements, in T1DM patients we observed significantly increased interventricular septal (IVS) and LV posterior wall (LVPW) thickness, but equal values of end diastolic LV dimension (LVIDd) and LVEF in comparison to healthy controls (Table 3).

Regarding right ventricular dimensions (RVD1, RVD1 index, RVD2, RVD3) and TAPSE we did not observe any significant difference in T1DM patients compared to healthy controls (Table 3).

Although mean levels of mitral and tricuspid PWD parameters were still within normal in both groups according to guidelines [14–16], mean peak late mitral (A) and tricuspid (At) inflow velocities were significantly increased in T1DM patients, while mitral (E/A) and tricuspid (Et/At) ratios were significantly decreased in T1DM patients in comparison to controls. Mitral DT was significantly prolonged in T1DM patients compared to control group (Table 3).

### TDI findings

TDI findings in T1DM patients compared to controls showed significantly decreased diastolic mitral annular velocities (E’sept, E’lat), significantly lower ratios E’/A’sept and E’/A’lat, significantly increased ratios E/E’sept, E/E’lat, significantly decreased S’sept, while the difference in S’lat was nonsignificant between T1DM patients and controls. LV IVA was significantly reduced in T1DM group compared to controls, while there was no significant difference in LV IVV between the groups (Table 3).

E’t was significantly decreased in T1DM patients while S’t was similar between the groups. The ratio E/E’t was significantly increased and E’/A’t was significantly decreased in diabetics in comparison to controls. RV IVV and RV IVA were both significantly reduced in diabetics compared to controls (Table 3).

We did not observe any significant difference in LV MPI and RV MPI between the groups (Table 3).

### Receiver operating characteristic curves analysis

**Fig. 1:**
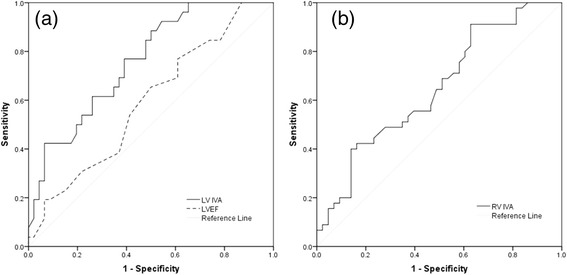
Receiver operating characteristic curves of **a** LV IVA and LVEF, **b** RV IVA. LV IVA, myocardial acceleration during isovolumetric contraction of the left ventricle; RV IVA, myocardial acceleration during isovolumetric contraction of the right ventricle; LVEF, left ventricular ejection fraction

ROC curves of systolic parameters (LV IVA, LVEF and RV IVA) are presented in Fig. 1. LV IVA provided the highest area under the curve (AUC) to detect early LV contractile dysfunction in diabetics (AUC 0.758, 95 % CI 0.648–0.869). Values of LV IVA below the cutoff point of 240 cm/s^2^ were characteristic for diabetics with 69.2 % sensitivity and 63.0 % specificity (*p* < 0.001). LVEF was not useful to predict early LV contractile impairement in diabetics (AUC 0.59, 95 % CI 0.455–0.725, *p* = 0.205). RV IVA below 295 cm/s^2^ was characteristic to detect early RV systolic dysfunction in diabetics (AUC 0.648, 95 % CI 0.533–0.763, *p* = 0.017), but sensitivity and specificity were very low (54.6 and 56.8 %, respectively).

**Fig. 2:**
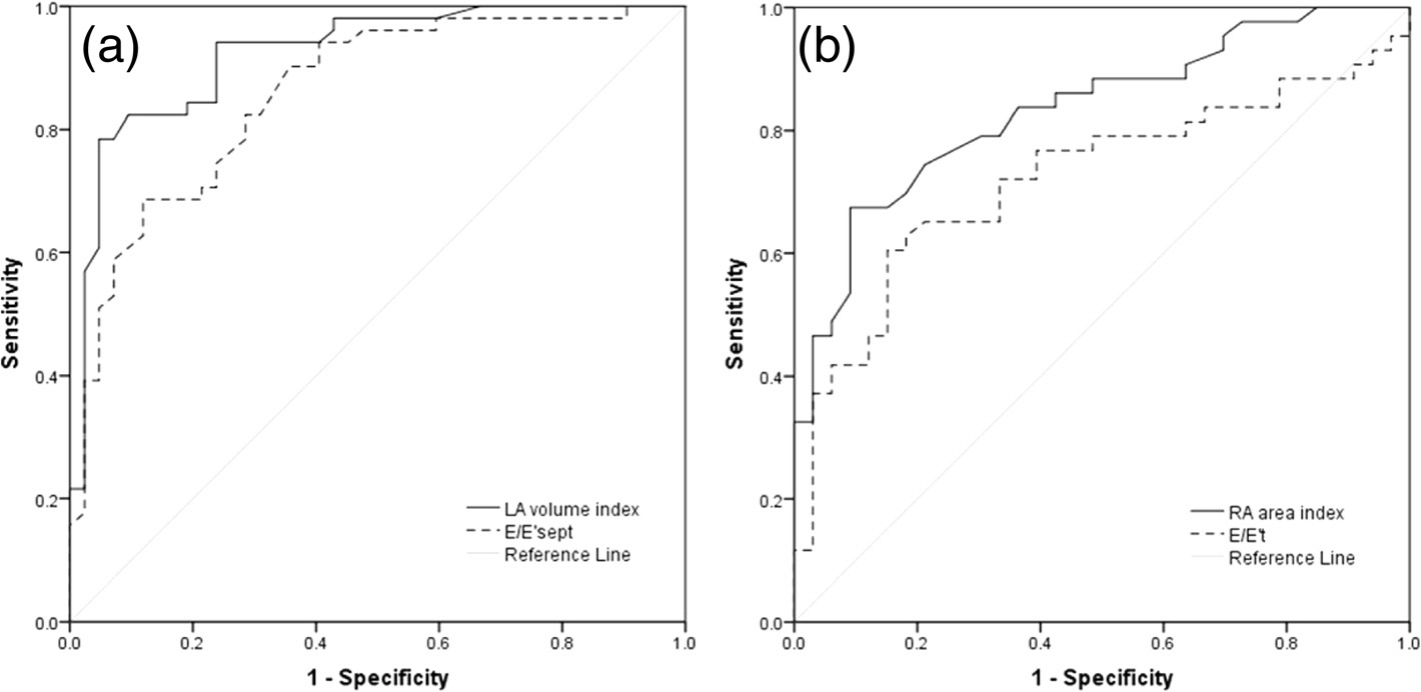
Receiver operating characteristic curves of **a** E/E’sept and LA volume index, **b** E/E’t and RA area index. LA, left atrium; RA, right atrium

ROC curves of diastolic parameters (E/E’sept, E/E’t, RA area index and LA volume index) are presented in Fig. 2. LA volume index and RA area index showed the highest AUC (AUC of LA volume index 0.923, 95 % CI 0.867–0.978, *p* < 0.001 and AUC of RA area index 0.833, 95 % CI 0.744–0.923, *p* < 0.001) to detect diabetics with early LV and RV diastolic impairement compared to healthy controls with the cutoff value of 26.0 ml/m^2^ for LA volume index (sensitivity 82.4 %, specificity 83.3 %) and 7.2 cm^2^/m^2^ for RA area index (sensitivity 74.4 %, specificity 78.8 %). Diagnostic accuracy of E/E’sept and E/E’t was also high (AUC of E/E’sept 0.857, 95 % CI 0.782–0.933, *p* < 0.001, AUC of E/E’t 0.722, 95 % CI 0.606–0.838, *p* = 0.001). Values above 7.4 for E/E’sept (sensitivity 76.5 %, specificity 73.8 %) and 3.4 for E/E’t (sensitivity 72.1 %, specificity 66.7 %) were characteristic for diabetic patients.

### Correlation analysis between LV / RV systolic function assessed by LV IVA/RV IVA and clinical, laboratory, echocardiographic parameters in diabetic patients 

In T1DM patients moderate but statistically significant correlations were found between LV IVA and E/E’lat (*r* = −0.38, *p* = 0.008) and S’lat (*r* = 0.316, *p* = 0.027). RV IVA correlated with BMI (*r* = 0.307), but the correlation met only borderline statistical significance (*p* = 0.045). No relationship was found between LV IVA and the following parameters: age, DM duration, BMI, E’sept, E’lat, E/E’sept, S’sept, S’t, E’t and E/E’t. Similarly, no correlation was found between RV IVA and age, DM duration, S’t, E’t, E/E’t, S’lat, S’sept, E/E’sept and E/E’lat. Furthermore, no correlations were confirmed between LV IVA/RV IVA and parameters of long-term glycemic control (HbA1c at enrollment and average HbA1c in the last 5 years). We found no correlation between LV IVA/RV IVA and other laboratory parameters (serum creatinine, total cholesterol, HDL, LDL, triglycerides, UACR) (Table 4).

**Table 4:** Correlation analysis between LV IVA/RV IVA and clical, laboratory and echocardiographic variables in diabetic patients

Variable	LV IVA (*n* = 53)		RV IVA (*n* = 53)	
	r	*p*-value	r	*p*-value
RV IVA	0.238	0.128		
Age (years)	−0.127	0.386	0.095	0.545
Duration of diabetes(years)	0.128	0.380	0.011	0.942
BMI (kg/m^2^)	0.112	0.444	0.307	0.045
HbA1C at enrollment (%)	0.078	0.632	0.068	0.695
HBA1c 5-year average (%)	0.034	0.835	0.124	0.472
Serum creatinine (μmol/L)	0.099	0.538	−0.008	0.964
Total cholesterol (mmol/L)	−0.116	0.476	0.122	0.477
Triglycerides (mmol/L)	0.023	0.890	0.094	0.585
HDL (mmol/L)	−0.063	0.698	−0,182	0.289
LDL (mmol/L)	−0.095	0.560	0.219	0.199
UACR (mg/g)	−0.272	0.103	−0.272	0.119
E’sept (cm/s)	−0.028	0.851	−0.228	0.142
E’lat (cm/s)	0.089	0.549	−0.129	0.417
E/E’sept	−0.191	0.189	−0.080	0.609
E/E’lat	−0.380	0.008	−0.199	0.207
S’sept (cm/s)	0.055	0.709	−0.014	0.927
S’lat (cm/s)	0.316	0.027	0.115	0.462
S’t (cm/s)	0.054	0.715	0.291	0.059
E’t (cm/s)	−0.065	0.659	0.135	0.388
E/E’t	0.027	0.855	−0.235	0.135

r- Pearson’s correlation coefficient

*IVA* myocardial acceleration during isovolumetric contraction, *BMI* body mass index, *HbA1c* glycosylated hemoglobin, *HDL* high density lipoprotein, *LDL* low density lipoprotein, *UACR* urinary albumin-to-creatinine ratio, *S’sept* peak systolic mitral annular velocity at the septal part of mitral annulus, *S’lat* peak systolic mitral annular velocity at the lateral part of mitral annulus, *E’sept* peak early diastolic mitral annular velocity at the septal part of mitral annulus, *E’lat* peak early diastolic mitral annular velocity at the lateral part of mitral annulus, *A’sept* peak late diastolic mitral annular velocity at the septal part of mitral annulus, *A’lat* peak late diastolic mitral annular velocity at the lateral part of mitral annulus, *S’t* peak systolic tricuspid annular velocity, *E’t* peak early diastolic tricuspid annular velocity, *A’t* peak late diastolic tricuspid annular velocity

### Observer variability

Intraobserver variabilities for LV IVV, LV IVA, RV IVV and RV IVA were 6.84 % (95 % CI 4.71–9.05), 7.22 % (95 % CI 5.46–9.22), 6.6 % (95 % CI 4.72–8.66) and 8.31 % (95 % CI 6.76–9.96), respectively. Interobserver variabilities for the corresponding measurements were 5.12 % (95 % CI 4.01–6.12), 11.54 (95 % CI 10.17–12.92), 5.05 % (95 % CI 4.11–5.96) and 9.70 % (95 % CI 8.49–10.82). ICC for intraobserver variability of LV IVV, LV IVA, RV IVV, RV IVA was 0.953 (95 % CI 0.871–0.983), 0.976 (95 % CI 0.933–0.992), 0.953 (95 % CI 0.872–0.983) and 0.958 (95 % CI 0.886–0.985), respectively. ICC for interobserver variabilty of the corresponding measurements was 0.987 (95 % CI 0.962–0.995), 0.946 (95 % CI 0.854–0.981), 0.986 (95 % CI 0.961–0.995) and 0.952 (95 % CI 0.868–0.983).

## Discussion

Our TDI results suggest that in T1DM patients diastolic function of both ventricles is impaired in comparison to matched healthy individuals and that there is a possible systolic impairement of both ventricles in T1DM patients as assessed by IVA.

Most of our mean PWD and TDI findings were in normal range for both groups according to current guidelines. However, small but statistically significant differences were detected in diabetics compared to healthy controls. E’sept, E’lat and E’t were decreased and E/E’sept, E/E’lat, E/E’t were all increased in diabetic group compared to controls, indicating worse diastolic function of both ventricles in this relatively young T1DM population. Mean LA volume index and mean RA area were in both groups still in normal range, but they were significantly higher in diabetics, suggesting long-term increase of LV and RV filling pressures in T1DM patients compared to healthy controls.

Recently, in a large observational echocardiographic study including 1091 T1DM patients (mean age 49.6 years, 53 % men) a high degree (30.8 %) of LV diastolic dysfunction has been documented among diabetics, which is more than expected in age-matched nondiabetic population [7]; similar prevalence of LV diastolic dysfunction was reported by Redfield et al. in more than 13 years older general population [17]. Some other studies including T1DM patients failed to detect LV diastolic impairement by PWD and confirmed superiority of TDI in evaluating subclinical LV diastolic abnormalities [11, 18]. In contrast to our findings a study of 185 normotensive T1DM patients and matched healthy controls from 2007 did not show any significant difference in LV diastolic function between the two groups neither by conventional echocardiography nor by TDI [6]. A recent study by Fagan et al. including patients with extremely long duration of T1DM – more than 50 years – surprisingly documented only slightly reduced E/A and elevated E/E’sept ratio compared to controls [19].

Since first published data on RV diastolic dysfunction in diabetic patients in 2004 [12], only few more studies have followed. Karamitsos et al. confirmed impaired RV diastolic function in T1DM patients by reduced tricuspid E/A ratio as well as by TDI analysis of E’t compared to controls, which is consistent with our results [10]. A study by Khattab et al. including diabetic children and adolescents demonstrated reduced E’sept and E’t as well as higher mitral and tricuspid E/E’ratios compared to matched controls, suggesting some degree of diastolic impairement of both ventricles in T1DM patients even at an early age [20].

Limited data exist on potential LV systolic dysfunction in T1DM patients. Most studies could not demonstrate LV contractile dysfunction by conventional echocardiography nor even by TDI [7, 10, 20].

Data on RV systolic function in T1DM are sparse. First research on RV contractile function in diabetics was published by Kosmala et al. in 2004 – they found no differences in color TDI-derived longitudinal shortening velocities of basal and mid segments of the RV free wall in a non-uniform diabetic group (including both T1DM and T2DM patients) compared to healthy controls [12]. Similarly, Karamitsos et al. found normal S’t assessed by pwTDI in T1DM patients [10].

A recent myocardial deformation study based on 2D speckle tracking echocardiography gave evidence of reduced global longitudinal strain of both ventricles in T1DM patients, while LV global radial strain and LV twist were not affected significantly [8]. This was in line with the hypothesis that left ventricular longitudinally oriented subendocardial fibers are the most vulnerable and susceptible to metabolic damage [8]. On the other hand Jensen et al. did not show the difference in LV longitudinal strain in the absence of albuminuria [21]. Interestingly, in the study by Fagan et al. including patients with more than 50 years of T1DM no difference was found in LV longitudinal and circumferential strain, strain rate and torsion [19].

Our TDI findings give additional evidence of impaired longitudinal contractility of the LV revealing significantly reduced S’sept and nonsignificantly reduced S’lat in diabetics. On the other hand we could not demonstrate reduced RV longitudinal shortening neither by TAPSE nor by S’t, which was consistent with previous reports [10]. This might be due to the different arrangement of RV myocardial fibers compared to the LV. The longitudinal RV shortening might be initially preserved in T1DM, while radial RV displacement has not been studied yet.

An important finding of our study is the potential of TDI-derived IVA to assess global systolic function of both ventricles in T1DM patients. In our study, IVA has proven to be the only useful TDI-derived parameter to evaluate potential early systolic impairement of both ventricles in T1DM patients, though sensitivity and specificity of RV IVA were low. At present we are not aware of any other study using this relatively novel TDI-derived parameter in T1DM population. A similar ability of IVA to detect LV systolic dysfunction was presented in a study including T2DM patients, whereas RV contractile dysfunction could not be confirmed by IVA in this study [22]. IVA was decreased in animal studies in diabetic rats compared to controls [23].

IVA is supposed to be a robust index of global contractility like dP/dt, as both indices reflect the change of contractile force during isovolumetric contraction [22]. IVA has been validated as a reliable and relatively load independent non-invasive measure of RV and LV systolic function [24–27]. It has been documented as a more sensitive parameter to assess LV contractility than TDI-derived peak systolic annular velocity [26]; It has been successfully applied for research purposes in different patient populations, including those with heart failure, valvular heart disease, chronic pulmonary artery disease and endocrine disorders [28, 29]. Recent studies have shown reliability of RV IVA in prediction of RV contractile dysfunction after heart valve surgery and its predictive role in diagnostics of acute pulmonary embolism [26, 30].

Our data show that in diabetics a moderate, but statistically significant positive correlation exists between LV IVA and S’lat - another parameter of LV contractility. Furthermore, LV IVA showed a moderate, but significant negative correlation with E/E’lat. This suggests that subclinical LV systolic and diastolic alterations might develop concurrently in T1DM patients, which has not been observed till now. However, we did not confirm these correlations for the RV.

### Study limitations

Considering ethical issues we did not perform coronary angiography to exclude coronary artery disease in our patients. However, based on detailed medical history all symptomatic patients with history of exertional angina or prior myocardial infarction were excluded from the study. Furthermore, we carefully excluded all patients with ischemic changes in the resting ECG, echocardiographic LV wall motion abnormalities and LVEF below 50 %. We believe that this limitation did not influence our results.

Baseline characteristics were not completely balanced, as diabetic patients had slightly elevated blood pressure compared to controls, but the difference was not statistically significant. As 24-h ambulatory blood pressure was not measured before enrollment, we cannot exclude potential masked hypertension in our patients. However, blood pressure was regularly checked in diabetic patients at our diabetes outpatient clinic and detailed medical hystory of home blood pressure measurements was taken in all participants before enrollment.

All echocardiographic measurements were performed by a single experienced echocardiographer who was not blinded to the diabetic status of participants. However, intraobserver and interobserver variabilities of IVV and IVA measurements were very good, suggesting adequate reproducibility of our results.

As exclusion criteria were very restrictive the number of diabetic patients was limited.

## Conclusions

PwTDI is a sensitive echocardiographic technique to detect subclinical LV and RV diastolic dysfunction in T1DM patients. Reduced IVA might be an early indicator of subclinical contractile (systolic) dysfunction of both ventricles in T1DM patients, though its diagnostic value is hampered by low sensitivity and specificity. Further research is warranted to compare the predictive value of pwTDI-derived LV IVA/RV IVA with 2D and 3D tissue deformation imaging to evaluate potential subclinical LV and RV contractile impairement in this subset of patients.

### Availability of data and materials

The dataset supporting the conclusions of this article is available in the »Figshare« repository (doi:10.6084/m9.figshare.3120979; https://figshare.com/articles/T1DM_echo_database_xlsx/3120979).

## Abbreviations

A: peak late mitral inflow velocity
A’lat: peak late diastolic mitral annular velocity at the lateral part of mitral annulus
A’sept: peak late diastolic mitral annular velocity at the septal part of mitral annulus
A’t: peak late diastolic tricuspid annular velocity
At: peak late tricuspid inflow velocity
BSA: body surface area
DT: deceleration time of peak early mitral inflow velocity
DT-t: deceleration time of peak early tricuspid inflow velocity
E: peak early mitral inflow velocity
E’lat: peak early diastolic mitral annular velocity at the lateral part of mitral annulus
E’sept: peak early diastolic mitral annular velocity at the septal part of mitral annulus
E’t: peak early diastolic tricuspid annular velocity
Et: peak early tricuspid inflow velocity
HbA1c: glycosilated hemoglobin
HDL: high density lipoprotein
IVA: myocardial acceleration during isovolumetric contraction
IVS: interventricular septum
IVV: peak myocardial velocity during isovolumetric contraction
LA: left atrium
LDL: low density lipoprotein
LV: left ventricle
LVEF: left ventricular ejection fraction
LVIDd: left ventricular internal dimension at end diastole
LVPW: left ventricular posterior wall
PWD: pulsed wave Doppler
pwTDI: pulsed wave tissue Doppler imaging
RA: right atrium
RV: right ventricle
RVD1: right ventricular basal diameter
RVD2: right ventricular mid-cavity diameter
RVD3: right ventricular longitudinal diameter
S’lat: peak systolic mitral annular velocity at the lateral part of mitral annulus
S’sept: peak systolic mitral annular velocity at the septal part of mitral annulus
S’t: peak systolic tricuspid annular velocity
TAPSE: tricuspid anular plane systolic excursion
UACR: urinary albumin-to-creatinine ratio
